# Dextrocardia in Heterotaxy Syndrome (Polysplenia Variant) in a 36‐Year‐Old Ethiopian Woman: A Case Report and Literature Review

**DOI:** 10.1155/cric/6419535

**Published:** 2026-06-18

**Authors:** Kedir Negesso Tukeni, Ramadan Jemal Dekema, Mohammed Kedir Shukri, Jafer Yasin Mohammed, Michael Deyu Jima, Merid Lemma Kebede

**Affiliations:** ^1^ Department of Internal Medicine, Jimma University, Jimma, Ethiopia, ju.edu.et; ^2^ Department of Radiology, Jimma University, Jimma, Ethiopia, ju.edu.et

**Keywords:** azygos continuation, case report, dextrocardia, Ethiopia, heterotaxy syndrome, left isomerism, polysplenia

## Abstract

**Background:**

Heterotaxy syndrome—polysplenia variant (left isomerism) with dextrocardia is a rare constellation of laterality defects characterized by left‐isomerism, multiple splenic nodules, and associated vascular and visceral anomalies. This report describes an adult Ethiopian woman who presented with nonspecific abdominal pain and was found to have imaging features diagnostic of heterotaxy (polysplenia variant).

**Case Presentation:**

A 36‐year‐old Ethiopian female presented with a three‐day history of mild to moderate, nonradiating anterior abdominal pain. Physical examination revealed the apical impulse on the right and mild left upper quadrant tenderness. Contrast‐enhanced CT of the chest and abdomen demonstrated dextrocardia with the cardiac apex directed to the right, right‐sided aortic arch, interrupted inferior vena cava with azygos continuation, bilobed lungs consistent with left isomerism, multiple splenic nodules clustered in the left upper quadrant (polysplenia), and a short pancreas. Routine laboratory tests were unremarkable. The final diagnosis was heterotaxy syndrome—polysplenia variant (left isomerism) with dextrocardia. The patient was managed conservatively for presumed costochondritis, counseled about her anatomical variation, advised to carry a medical alert card, and remained symptom‐free at two‐week follow‐up.

**Conclusion:**

Imaging features in this patient are most consistent with heterotaxy syndrome (polysplenia variant) rather than isolated situs inversus totalis. Clear, consistent diagnostic terminology (heterotaxy—polysplenia variant with dextrocardia) is essential for accurate communication, appropriate counseling, and safe planning of future interventions. Recognition of such cases in underreported regions supports improved diagnostic awareness and tailored care.

**Key Clinical Messages**

• Diagnosis: Heterotaxy syndrome**—**polysplenia variant with dextrocardia (left isomerism) is distinct from situs inversus totalis.
• Imaging: Precise cross‐sectional imaging is essential to define organ and vascular anatomy.
• Cardiac work‐up: Perform ECG, chest radiograph, and transthoracic echocardiography to exclude congenital heart disease.
• Spleen: Anatomic polysplenia is not functional hyposplenism; assess splenic function before asplenia‐level interventions.
• Infection prevention: If hyposplenism is confirmed, follow local guidance for vaccines and consider prophylaxis; otherwise, counsel on fever urgency.
• Perioperative care: Document and communicate mirror‐image and vascular anomalies for procedural planning.
• Patient advice: Provide a medical alert card and arrange cardiology and, if indicated, splenic‐function follow‐up.

## 1. Introduction

Dextrocardia with situs inversus and polysplenia constitutes a rare constellation of congenital anomalies characterized by mirror‐image reversal of thoracic and abdominal organs, frequently accompanied by multiple spleens [1–3]. Although each anomaly may occur independently, their coexistence presents distinctive diagnostic, clinical, and management challenges. Globally, the prevalence of these conditions is low, and data regarding their epidemiology and clinical implications in African populations, particularly in Ethiopia, remain scarce [2, 4–6]. This report describes a 36‐year‐old Ethiopian woman who presented with nonspecific abdominal pain and was incidentally found to have dextrocardia, situs inversus, and polysplenia. The case is further contextualized through a comprehensive review of the literature, addressing epidemiology, embryological and genetic mechanisms, clinical significance, diagnostic approaches, management strategies, and outcomes, with emphasis on both African and global perspectives.

The final diagnosis was dextrocardia with heterotaxy syndrome (polysplenia variant) and polysplenia, identified incidentally during the evaluation of nonspecific abdominal pain. The pain was ultimately attributed to costochondritis, and the patient was managed conservatively with acetaminophen and reassurance. She received comprehensive counseling regarding her unique anatomical configuration, its potential implications for future medical care, and the importance of alerting healthcare providers in emergency or surgical settings. To enhance safety, she was advised to carry a medical alert card and was scheduled for follow‐up. At the two‐week review, her symptoms had completely resolved, and she remained clinically well.

### 1.1. Literature Review Methods

We performed a focused literature search to identify reports and reviews of dextrocardia, situs anomalies, and polysplenia. Searches were run in PubMed/MEDLINE, Embase, and Google Scholar for articles published up to April 2026 using combinations of the terms “dextrocardia,” “situs inversus,” “heterotaxy,” “polysplenia,” “left isomerism,” and “azygos continuation.” We included case reports, case series, and review articles in English and excluded animal studies and conference abstracts without full text. Titles and abstracts were screened for relevance, and full texts were reviewed when available; references of selected articles were hand‐searched for additional reports. Data were synthesized narratively because the heterogeneity of case descriptions and imaging findings precluded quantitative pooling. We report the search strategy and selection approach to provide transparency and to contextualize the case within existing literature.

## 2. Case Presentation

A 36‐year‐old Ethiopian female from Jimma, Ethiopia, presented to the outpatient department of medicine at a tertiary hospital with a three‐day history of dull, intermittent anterior abdominal pain. The pain was mild to moderate in severity, nonradiating, and not associated with vomiting, fever, or epigastric discomfort. She denied any history of trauma, previous abdominal surgery, diabetes, chronic lung disease, or similar symptoms in the past. This was her first presentation to a healthcare facility. Her family history was noncontributory, with no known congenital anomalies or similar conditions among relatives. She reported no significant psychosocial stressors or exposures during pregnancy, and there was no history of consanguinity. On presentation, the patient′s vital signs were: temperature 36.8°C, heart rate 78 bpm, blood pressure 118/72 mmHg, respiratory rate 16 brpm, and oxygen saturation 98% on room air. A 12‐lead ECG showed sinus rhythm at 86 bpm with rightward axis consistent with dextrocardia and no ischemic ST‐T changes; lead placement was confirmed. Chest radiograph demonstrated the cardiac apex on the right without acute airspace consolidation. Transthoracic echocardiography showed normal chamber sizes, preserved biventricular systolic function (LVEF ~63%), and no intracardiac shunt or significant valvular abnormality. Laboratory tests were within normal limits: hemoglobin 13.2 g/dL (ref 12–16), WBC 6.4 × 10^9^/L (ref 4–11), platelets 280 × 10^9^/L (ref 150–400), CRP < 5 mg/L (ref < 5), creatinine 0.8 mg/dL (ref 0.6–1.2), AST 22 U/L (ref 10–40), ALT 18 U/L (ref 7–56), lipase 35 U/L (ref 0–60). Blood cultures were not obtained.

The patient′s abdominal and anterior chest pain was judged most consistent with a musculoskeletal chest‐wall process (working diagnosis: costochondritis) because focal tenderness was reproducible on palpation of the anterior chest wall, there were no peritoneal signs on abdominal examination, inflammatory markers and pancreatic enzymes were within institutional reference ranges, and cross‐sectional imaging showed no acute intra‐abdominal pathology. The pain responded to a short course of acetaminophen with symptomatic resolution at two‐week follow‐up. Given these findings, a musculoskeletal etiology was considered most likely; however, other causes (including biliary pathology, splenic complications, pancreatitis, and intestinal malrotation‐related complications) were considered and monitored for during follow‐up.

**Figure 1:**
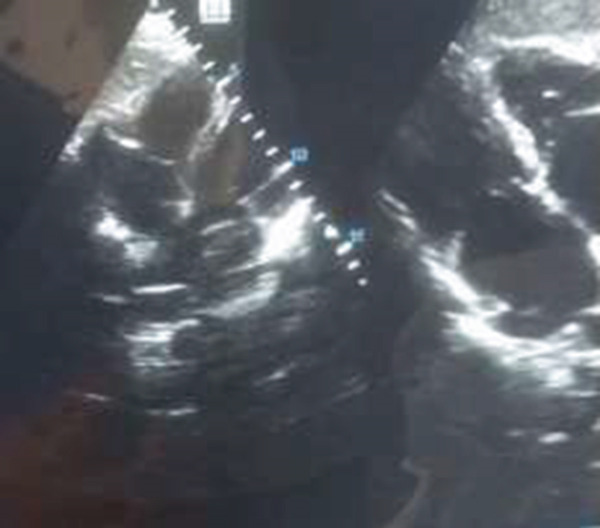
Transthoracic echocardiography of the patient in subcostal four‐chamber view showing normal cardiac chambers with apex directed towards the right of the patient′s chest.

Cardiac auscultation revealed normal S1 and S2 heart sounds, but the apical impulse was palpated in the right fifth intercostal space along the midclavicular line (Figure 1: The white arrow shows the apex of the heart on the right side of the chest). No murmurs or additional heart sounds were detected.

Abdominal ultrasound shows that the liver and gallbladder are located in the left upper quadrant, the stomach and multiple splenic nodules (poly splenia) are also located in the left upper quadrant. The inferior vena cava is located on the left side, whereas the abdominal aorta is on the right side, with a nonvisualized intrahepatic segment of the inferior vena cava, which is continued as azygos continuation of IVC.

**Figure 2:**
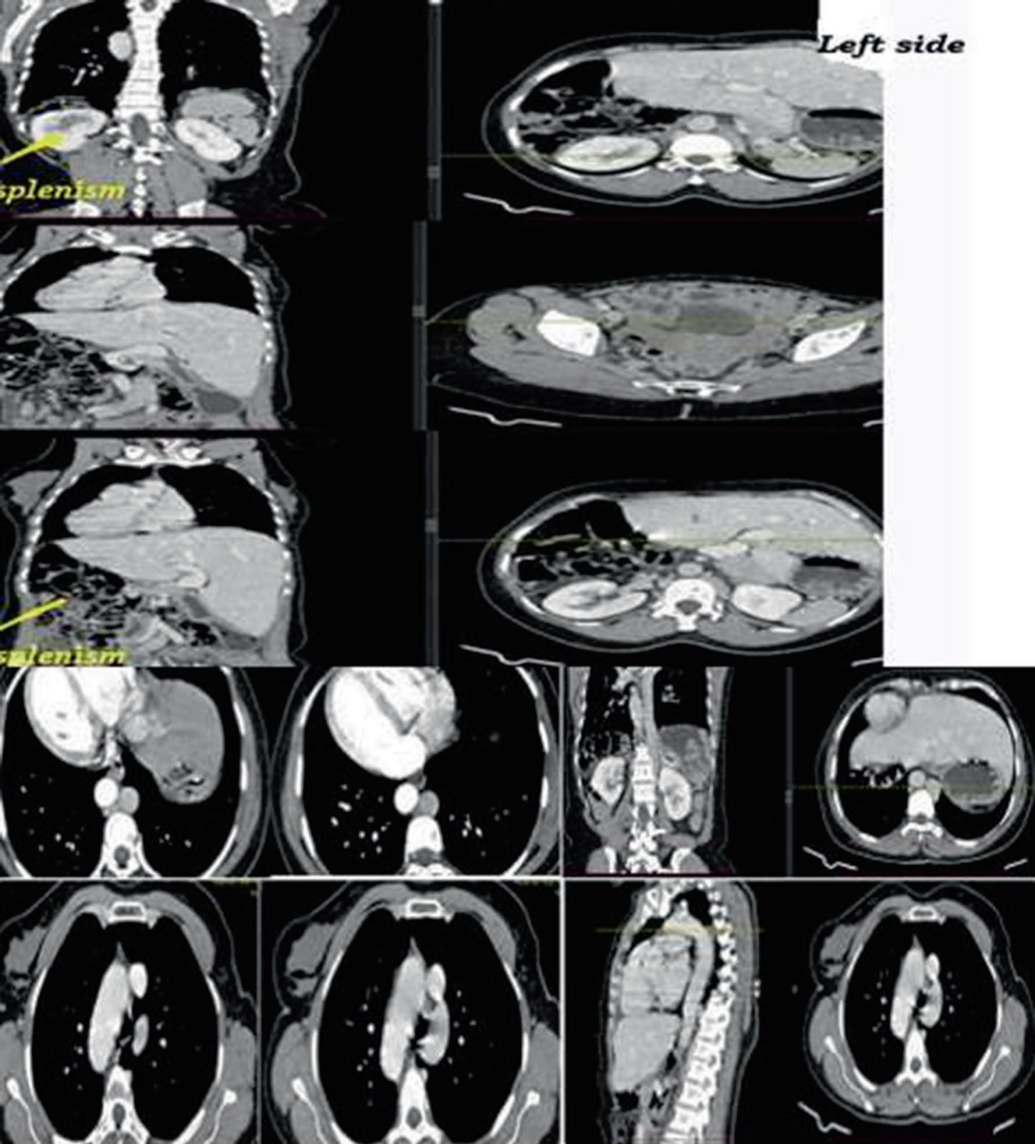
Contrast‐enhanced CT of the chest and abdomen demonstrates multiple anomalies consistent with heterotaxy syndrome, polysplenia type (Figure 2: yellow arrows). The heart is located in the right hemithorax with the apex directed to the right (dextrocardia). A right‐sided aortic arch is present, and the inferior vena cava shows azygos continuation. She has also bilaterally bilobed lungs (left isomerism). In the abdomen, the liver and gallbladder are situated on the left, the stomach is left‐sided, and multiple splenic nodules are clustered in the left upper quadrant, consistent with polysplenia. The pancreas appears short, whereas the portal venous anatomy remains normal. Collectively, these findings indicate dextrocardia with heterotaxy syndrome (polysplenia variant), right‐sided aortic arch, azygos continuation of the IVC, and polysplenia, diagnostic of heterotaxy syndrome (polysplenia variant).

Contrast‐enhanced CT of the chest and abdomen demonstrated features diagnostic of heterotaxy syndrome—polysplenia variant (left isomerism): the heart was located in the right hemithorax with the apex directed to the right (dextrocardia); a right‐sided aortic arch was present; the inferior vena cava was interrupted with azygos continuation; the lungs showed bilobed morphology consistent with left isomerism; multiple splenic nodules were clustered in the left upper quadrant (polysplenia); and the pancreas appeared short whereas portal venous anatomy remained preserved. Routine laboratory tests and inflammatory markers were within normal limits (Figure 2), and Video 1).

The remainder of the systemic examination was unremarkable, with no evidence of cyanosis, clubbing, or peripheral edema. The summary of the evaluation revealed dextrocardia and heterotaxy syndrome (polysplenia variant), along with polysplenia and a short pancreas, confirmed through imaging studies. Routine laboratory tests and inflammatory markers were normal, with no signs of infection or acute pathology.

Final diagnosis: heterotaxy syndrome—polysplenia variant (left isomerism) with dextrocardia, interrupted IVC with azygos continuation, right‐sided aortic arch, multiple splenic nodules, and short pancreas.

## 3. Management and Follow‐up

### 3.1. Splenic Function Assessment

Although CT demonstrated multiple splenic nodules consistent with polysplenia, splenic function was not assumed to be impaired. However, a peripheral blood smear was not examined for Howell‐Jolly bodies (HJBs), nor was pitted red cell testing. In the absence of laboratory or scintigraphic evidence of hyposplenism, no routine antibiotic prophylaxis was started.

### 3.2. Vaccination and Prophylaxis

Because anatomical polysplenia alone does not confirm functional hyposplenism, vaccination against encapsulated organisms (pneumococcus, meningococcus, Hib) was not recommended. The patient was counseled to seek urgent care for fever and to carry a medical alert card indicating heterotaxy with polysplenia.

## 4. Discussion

Heterotaxy syndrome encompasses a spectrum of laterality defects in which normal left–right organ asymmetry is disrupted. The polysplenia variant (left isomerism) is characterized by features of bilateral left‐sidedness, including multiple splenic nodules, bilobed lungs, and frequent vascular anomalies such as interrupted inferior vena cava with azygos continuation. By contrast, situs inversus totalis denotes a complete mirror‐image arrangement of thoracic and abdominal organs without isomerism or the vascular interruptions typical of heterotaxy. In our patient, the combination of multiple splenic nodules, bilobed lungs, interrupted IVC with azygos continuation, and a short pancreas supports the diagnosis of heterotaxy syndrome—polysplenia variant (left isomerism) with dextrocardia, rather than isolated situs inversus totalis. Using precise terminology clarifies prognosis, guides screening for associated anomalies, and informs perioperative planning.

Dextrocardia with heterotaxy syndrome (polysplenia variant) is a rare congenital anomaly, with a global prevalence estimated between 1 in 8000 and 1 in 20,000 live births [5, 7–9]. Although situs inversus totalis denotes complete mirror‐image organ arrangement, heterotaxy (polysplenia variant) is characterized by left‐isomerism and associated vascular anomalies (e.g., interrupted IVC with azygos continuation)—features present in our patient and therefore more consistent with heterotaxy than with isolated situs inversus totalis. The condition affects males and females equally, with no significant variation by ethnicity. Even rarer is polysplenia, a manifestation of left isomerism within the heterotaxy spectrum, with an estimated incidence of 1 in 250,000 live births [5, 9]. Data from African populations remains limited, though several case reports from Ethiopia and other countries have documented similar presentations. These reports suggest that such anomalies are likely underrecognized and underdiagnosed, largely due to restricted access to advanced imaging and genetic testing [5, 8, 9].

The establishment of left–right asymmetry during embryogenesis is a highly regulated process involving molecular signaling cascades and the coordinated motion of motile cilia at the embryonic node. These cilia generate directional fluid flow, which activates the Nodal signaling pathway and downstream transcriptional regulators that determine organ laterality. Disruption of these pathways—whether through genetic mutations or impaired ciliary function—can result in laterality defects such as situs inversus or heterotaxy syndromes. Genes commonly implicated include those encoding ciliary dynein arms (e.g., *DNAH5*, *DNAI1*) and other regulators of ciliary assembly and motility [10–14].

Heterotaxy syndrome (polysplenia variant) represents a complete mirror‐image reversal of thoracic and abdominal organs, including the heart, liver, stomach, and spleen. Although often clinically silent, it is usually inherited in an autosomal recessive pattern, though X‐linked and sporadic cases have been reported. In contrast, polysplenia syndrome (left isomerism) is characterized by multiple spleens and bilateral left‐sidedness of thoracic and abdominal structures. It is frequently associated with complex vascular and visceral anomalies, such as interrupted inferior vena cava with azygos continuation, intestinal malrotation, and congenital cardiac malformations, which may complicate clinical management [11, 12, 15–17].

Defective ciliary motility during embryogenesis can randomize organ positioning, leading to syndromes such as primary ciliary dyskinesia (PCD). In this disorder, impaired ciliary function disrupts left–right signaling, and approximately 50% of affected individuals exhibit situs inversus. When combined with chronic sinusitis and bronchiectasis, this presentation is termed Kartagener syndrome. Patients with PCD often experience recurrent respiratory infections, progressive airway disease, and, in males, infertility due to immotile sperm [18–20]. These observations highlight the central role of ciliary biology in establishing normal organ asymmetry and underscore the wide clinical spectrum of its disruption, ranging from benign anatomical variants to life‐threatening congenital anomalies [17–21].

Most individuals with isolated heterotaxy syndrome (polysplenia variant) remain asymptomatic and are diagnosed incidentally during imaging for unrelated conditions. However, the mirror‐image arrangement of organs can complicate the evaluation of common diseases—for example, appendicitis may present with left lower quadrant pain, and cholecystitis may manifest with left upper quadrant pain. Beyond situs inversus, heterotaxy syndromes such as polysplenia carry greater clinical significance, with congenital heart disease present in more than half of cases, though generally less severe than in asplenia (right isomerism). These complex anatomical variations highlight the importance of careful imaging, multidisciplinary evaluation, and individualized management strategies, particularly in resource‐limited settings where diagnostic challenges are amplified (Table 1) [19, 20, 22–31].

**Table 1:** Distinguishing features of dextrocardia with situs inversus and polysplenia. Although both may be asymptomatic, polysplenia carries a higher risk of congenital heart disease, vascular anomalies, and functional hyposplenism, requiring tailored management.

Characteristics	Dextrocardia with situs inversus	Polysplenia syndrome (left isomerism)
Prevalence	1 in 8000–20,000 live births	1 in 250,000 live births
Gender predilection	None	More common in females
Inheritance	Autosomal recessive/X‐linked	Multifactorial, genetic, teratogenic
Associated cardiac anomalies	3%–5% (isolated cases)	> 50%, usually noncyanotic, less severe
Splenic findings	Single spleen, right‐sided	Multiple spleens, variable location
Vascular anomalies	Rare	Interrupted IVC, azygos continuation, PDPV
GI anomalies	Rare	Midgut malrotation, short pancreas
Clinical presentation	Often asymptomatic	Variable, depends on associated anomalies
Infection risk	Normal (if spleen is functional)	Increased (functional hyposplenism possible)
Management	Supportive, educational	Vaccination, infection prophylaxis, and surgery
Prognosis	Excellent (isolated cases)	Depends on cardiac and splenic function

Dextrocardia with situs inversus and polysplenia represents a rare but clinically important constellation of congenital anomalies that is often asymptomatic and discovered incidentally, yet poses significant challenges for diagnosis, imaging interpretation, and surgical management due to reversed anatomy [2, 3, 32]. Polysplenia syndrome is frequently associated with vascular and visceral anomalies, and functional hyposplenism may heighten susceptibility to severe infections, underscoring the need for vaccination and prophylactic measures [1, 2, 32, 33]. Awareness of these conditions is essential for clinicians, radiologists, and surgeons to prevent misdiagnosis and ensure safe care, whereas genetic counseling, patient education, and adherence to ethical reporting standards remain integral to long‐term management. Prognosis is generally favorable in isolated cases, but outcomes depend on the severity of associated anomalies, particularly congenital heart disease and splenic dysfunction [2, 32]. Greater research and case documentation from Africa and other underrepresented regions are needed to clarify epidemiology, clinical spectrum, and best practices for management. In patients with situs inversus and polysplenia, abdominal pain may be linked to associated intestinal malrotation, a condition that can remain silent until adulthood but predisposes to complications such as volvulus [34]. In our case, this possibility underscores the need for careful radiologic evaluation. Moreover, Ethiopian data show congenital anomalies contribute significantly to morbidity and mortality, with limited access to advanced imaging and surgical care often delaying diagnosis [35]. This context highlights the importance of early recognition and tailored management strategies to reduce risks compared to higher‐resource settings.

## 5. Conclusion

This case represents heterotaxy syndrome—polysplenia variant (left isomerism) with dextrocardia, confirmed by CT imaging showing multiple splenic nodules, bilobed lungs, interrupted IVC with azygos continuation, right‐sided aortic arch, and a short pancreas. Precise diagnostic labeling as heterotaxy (polysplenia variant) is important because it reflects the presence of isomerism and vascular anomalies that differ from isolated situs inversus totalis and that have implications for infection risk, cardiac evaluation, and procedural planning. In the absence of significant cardiac or hemodynamic compromise, conservative management and patient education were appropriate; however, clinicians should remain vigilant for associated anomalies and counsel patients to inform future healthcare providers of their diagnosis.

## Author Contributions

All authors made a significant contribution to the work reported, whether that is in the conception, study design, execution, acquisition of data, analysis and interpretation, or in all these areas; took part in drafting, revising or critically reviewing the article; gave final approval of the version to be published; have agreed on the journal to which the article has been submitted; and agree to be accountable for all aspects of the work.

## Funding

No funding was received for this manuscript.

## Ethics Statement

Ethical approval was unnecessary, but participants′ information was kept confidential and used exclusively for this publication without identifying patients.

## Consent

Written informed consent was obtained from the patient for the publication of this case report and images, available for review by the editor‐in‐chief.

## Conflicts of Interest

The authors declare no conflicts of interest.

## Supporting information


**Supporting Information** Additional supporting information can be found online in the Supporting Information section. Video 1: Contrast‐enhanced CT of the chest and abdomen demonstrates multiple anomalies consistent with heterotaxy syndrome, polysplenia type. The heart is located in the right hemithorax with the apex directed to the right (dextrocardia). A right‐sided aortic arch is present, and the inferior vena cava shows azygos continuation. In the abdomen, the liver and gallbladder are situated on the left, the stomach is left‐sided, and multiple splenic nodules are clustered in the left upper quadrant, consistent with polysplenia. The pancreas appears short, whereas the portal venous anatomy remains normal. Collectively, these findings indicate dextrocardia with heterotaxy syndrome (polysplenia variant), right‐sided aortic arch, azygos continuation of the IVC, and polysplenia, diagnostic of heterotaxy syndrome (polysplenia variant).

## Data Availability

The data that support the findings of this study are available from the corresponding author upon reasonable request.
